# Optimization of Atmospheric Cold Plasma Treatment with Different Gases for Reduction of *Escherichia coli* in Wheat Flour

**DOI:** 10.4014/jmb.2203.03056

**Published:** 2022-04-15

**Authors:** Jeongmin Lee, Seul-Ki Park, Darren Korber, Oon-Doo Baik

**Affiliations:** 1 Department of Chemical and Biological Engineering, University of Saskatchewan, Saskatoon, SK S7N 5A9, Canada; 2 Department of Food and Bioproduct Sciences, University of Saskatchewan, Saskatoon, SK S7N 5A8, Canada

**Keywords:** Non-thermal treatment, atmospheric cold plasma, wheat flour, response surface method, *Escherichia coli*

## Abstract

In this study we aimed to derive the response surface models for *Escherichia coli* reduction in wheat flour using atmospheric cold plasma (ACP) with three types of gas. The jet-type atmospheric cold plasma wand system was used with a 30 W power supply, and three gases (argon, air, and nitrogen) were applied as the treatment gas. The operating parameters for process optimization considered were wheat flour mass (g), treatment time (min), and gas flow rate (L/min). The wheat flour samples were artificially contaminated with *E. coli* at a concentration of 9.25 ± 0.74 log CFU/g. ACP treatments with argon, air, and nitrogen resulted in 2.66, 4.21, and 5.55 log CFU/g reduction of *E. coli*, respectively, in wheat flour under optimized conditions. The optimized conditions to reduce *E. coli* were 0.5 g of the flour mass, 15 min of treatment time, and 0.20 L/min of nitrogen gas flow rate, and the predicted highest reduction level from modeling was 5.63 log CFU/g.

## Introduction

Wheat flour had not drawn attention in terms of food safety as the cooking process inactivates most of the pathogenic bacteria in wheat flour. However, concerns were raised after outbreaks between 2015-2017 in Canada and the USA of the shiga toxin-producing *Escherichia coli* (STEC) O121 and O26, caused by consumption of uncooked baking flour [1-3]. According to Gill *et al*. [2], the naturally contaminated STEC O121 in wheat flour survived two years or more in storage at room temperature. Meanwhile, *E. coli* contamination in wheat flour has been reported in other countries as well, including Turkey and Australia [2, 4]. Following two reports of *E. coli* outbreaks linked to wheat flour, the Canadian Food Inspection Agency (CFIA) recommended that the product should not be consumed raw [5]. Apart from appropriate food safety practices expected from consumers, wheat flour manufacturers take pains to control pathogenic microbes from the production stage.

Various treatment methods (heat, irradiation, and ozone) have been reported to reduce microbial contamination in wheat flour [6-8]. Li *et al*. [7] measured the microbial growth that occurred in fresh noodle dough using microwave heat-treated wheat flour at 2,000 W for 40 s. The time before the total microbial population reached 6 log CFU/g from freshly made noodle dough was extended from 12 to 48 h with the microwave treatment. The authors also determined that microwave treatment altered the farinograph and extensograph parameters of wheat flour. Agundez-Arvizu *et al*. [6] studied gamma irradiation treatment of Mexican bread wheat flour and the associated nutritional, rheological, and microbial changes that occurred post-treatment. They found that the rheological and nutritional properties had not been significantly changed, while the total aerobic counts of the wheat flour were reduced by 96% with irradiation treatment. Liu *et al*. [8] examined the microbial impact of ozone treatment on wheat bran, which reduced 90% of the viable microbial load from the product and extended the shelf life of noodle dough freshly made from wheat flour with the addition of ozone-treated wheat bran. Thus, ozone is effective for deactivating bacteria, but when its concentration reaches a critical level, it may react with other reactive agents to produce additional unpredicted compounds. While treatments such as these have succeeded in reducing microorganisms in flours, there is still potential for further improvements as these antimicrobial treatments are insufficient to fully inactivate the targeted microorganisms. For example, atmospheric cold plasma (ACP) produces various reactive radicals by the carrier gas so that microorganisms can be inactivated through several mechanisms.

ACP has attracted attention as one of the more promising non-thermal technologies for the reduction of viable bacteria in difficult-to-treat foods such as dry flours. Plasma refers to a partially or entirely ionized gas composed of free electrons, photons, and ions [9]. Additionally, plasma is composed of excited or fundamental states of atoms with a net neutral charge [10]. ACP is primarily known to have a bactericidal effect with low collateral impact on the sample. [11]. Treatment with ACP generates reactive oxygen species (ROS) groups and reactive nitrogen species (RNS), which produces an anti-microbial effect [12]. These ROS and RNS are associated with bactericidal effect by permanent cell wall destruction that leads to leakage of intracellular compounds such as DNA, proteins and lipids [13]. Due to these advantages, ACP has been investigated for application to reduce the viable microbial load in food products and pharmaceutical packaging materials [12, 14-16].

In particular, several studies proved that ACP application on wheat flour altered rheological parameters, but showed limited microbial reduction ability due to insufficient period of application [17, 18]. We therefore sought to design a way to improve the bactericidal effect of ACP application for wheat flour through the structural improvement of the treatment device and optimization of important parameters for ACP treatment of wheat flour using the response surface method.

## Materials and Methods

### Sample Preparation of Wheat Flour Inoculated with *E. coli*

All-purpose wheat flour (Smucker Foods of Canada Corp., Canada) was purchased at a local market. The pre-inoculated flour sample was stored in a sealed container at -20°C for subsequent experiments. *Escherichia coli* ATCC 14763 was obtained from the American Type Culture Collection, grown according to ATCC instructions, and stored in 20% (w/v) glycerol at -80°C prior to experimental use. Thawed *E. coli* inoculant was transferred into full-strength tryptic soy broth (TSB, BD Difco, USA) and incubated at 37°C for 24 h with constant shaking at 200 rpm. The cultured cells were centrifuged at 3,220 ×*g* for 15 min at 4°C, and the pellets were washed twice with sterile saline water (0.90% NaCl, w/v). A 5 ml volume of 10% sucrose solution was added to the washed pellets and stored overnight at -80°C prior to freeze-drying the inoculum using a Free Zone 6-L Stoppering Tray Dry System (Labconco Corporation, USA) at -50°C for 50 h. The obtained freeze-dried powder of the inoculant (~30 mg) was stored at -20°C. For the preparation of contaminated wheat flour by *E. coli*, 30 g of flour and freeze-dried inoculant were placed in a sterile stomacher bag (Seward Laboratory Systems Inc., USA). Mixing of wheat flour and inoculants was completed by a vortex shaker (Labnet Inc., USA) for 5 min or more until a uniform mixture was observed.

### Atmospheric Cold Plasma Setup

**Fig. 1:**
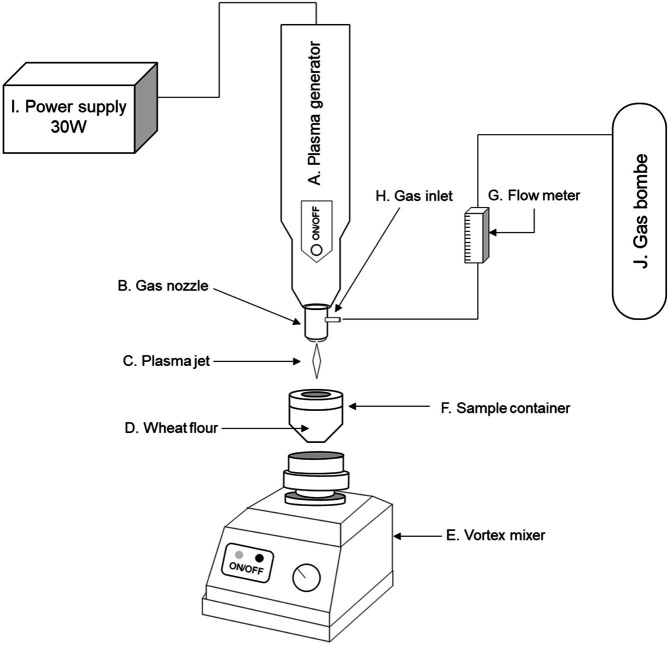
Schematic diagram of the atmospheric cold plasma (ACP) treatment system with carrier gas. **A**, plasma generator; **B**, gas nozzle; **C**, plasma jet; **D**, wheat flour sample; **E**, vortex mixer; **F**, customized sample treatment container; **G**, gas flow meter; **H**, gas inlet; **I**, power supply; **J**, gas bombe.

The ACP equipment used in this study is shown in Fig. 1. A certain amount (0.5 to 1.5 g) of wheat flour was transferred into a 9.5-ml customized polypropylene container. The distance between the top and bottom of the customized container was 30 mm. Also, the distance from the flour surface to the tip of the plasma wand was 10-20 mm. The container was placed on a vortex mixer (Labnet Inc., USA) and the flour particles were continuously vibrated at 3,400 rpm for efficient contact between the flour particles and the plasma. The gas nozzle connected to the gas cylinder through the gas flow meter was connected to the 30 W plasma generator (Plasma Etch Inc., USA) that was fixed to the retort stand. The temperature of the ACP was under 50°C, and the atmospheric conditions were 20 ± 2°C and 22 ± 2% relative humidity, as measured by a hygrometer.

### Microbiological Analysis

The reduction level of *E. coli* was determined from the difference between viable cell counts (log CFU/g) of non-treatment (control) and after-treatment flour. The wheat flour samples of control and after-treatment were weighed and mixed with 9 times the volume of saline water (0.90%, w/v). Then, the samples were serially diluted to an appropriate level, spread-plated on tryptic soy agar (TSB, BD Difco, USA), and incubated at 37°C for 24 h.

### Response Surface Methodology (RSM) Experimental Design

Three factors were selected for investigation of their interactions to optimize *E. coli* reduction in wheat flour with ACP treatment using the Design-Expert 13.0.2 software package (Stat-Ease Inc., USA). Three experimental designs were employed and consisted of different carrier gases that included argon (≥ 99.9%), air (O_2_ concentration 20-22%), and nitrogen (≥ 99.9%) to compare the reduction effectiveness among gas types. Table 1 shows the chosen parameters: mass of wheat flour (*x*_1_), treatment time (*x*_2_), and gas flow rate (*x*_3_). The flour samples (0.5, 1.0, and 1.5 g) were treated for three durations (5, 10, and 15 min) through various gas flow rates (0.04, 0.12, and 0.20 L/min) using the three gases (argon, air, and nitrogen). The reduction of *E. coli* (log CFU/g, Y) was selected as a response parameter (dependent variable) for modeling with the three gases. The Box-Behnken design (BBD) is an independent, rotatable quadratic design that consists of a central point with the middle points of the edges of the cube circumscribed on a sphere [19]. This approach has previously been applied to study the effect of process variables [20]. The BBD in the Design-Expert program suggested 17 runs of the experiment be made for each gas, including five replicates at the central point in order to allow the estimation of pure error. The method assumes that the experimental errors from the central points are equally distributed to all the combinations. The correlation between the independent ACP treatment parameters and the response were described by a quadratic polynomial response equation:

$$Y=\beta_{0}+\sum {}_{i=1}^{k}\beta_{i}x_{i}+\sum {}_{i=1}^{k}\beta_{ii}x_{i}^{2}+\sum {}_{i=1}\sum {}_{j>i}^{k}\beta_{ij}x_{i}x_{j}+\varepsilon ,$$ (1)

where x_i_ and x_j_ are the independent variables and k is the number of variables. Y is the predicted response. b_0_, b_i_, b_ii_, and b_ij_ are constant, linear, quadratic, and interaction coefficients, respectively. Lastly, e is the random error [21].

### Validation of the Developed Models

The validation of the regression models was performed for the optimized conditions determined by the Design-Expert program. The validation experiment was conducted for each gas. The actual combination used in the validation was determined to be the closest integer to the derived values. The predicted responses and actual responses were compared through analysis of variance (ANOVA) at the confidence level of 95% (*p* < 0.05).

### Statistical Analysis

The average and standard deviations of the results were calculated based on triplicate experiments. The statistical analysis was conducted using ANOVA and Tukey’s multiple comparisons with the PRISM 9.3.1 (GraphPad Software, USA) at the confidence level of 95% (*p* < 0.05). The significant *p*-value for ANOVA analysis and insignificant lack-of-fit value validated the significance of the model. The model equations were derived and examined after the statistical analysis.

## Results and Discussion

### Bactericidal Effect of ACP Treatment with Three Gases

The results of ACP treatment with three types of gas according to 17 runs suggested by BBD are shown in Table 2. The initial contamination level of artificially inoculated wheat flour was 9.25 ± 0.74 log CFU/g. The results are given as a reduction of log values of *E. coli* in the wheat flour. No bactericidal effect was observed in a few runs with argon and air. Runs no. 4, 9, 10, and 11 using argon, and run no. 2 using air did not show any bactericidal effect. However, all the runs with nitrogen gas showed a bactericidal effect. The minimum and maximum reductions by ACP with nitrogen gas were 1.01 log CFU/g and 4.94 log CFU/g, respectively. ACP with nitrogen gas was the most effective for reducing *E. coli* among the three types of gases. ACP with nitrogen gas produces a certain amount of RNS, such as NO, N_2_, and NO_3_, among the main effective species. Therefore, ACP treatment with nitrogen gas could exhibit greater bactericidal effect. The maximum reduction by ACP with argon was 2.65 log CFU/g, while in the case of ACP with air, it was 3.85 log CFU/g. According to gas type, bactericidal effect was rated in the order of nitrogen > air > argon. This result was supported by Feichtinger *et al*. [22]. They also reported a bactericidal effect using different gases such as air, argon, and ammonia for the plasma treatment, with air showing the highest bactericidal effect and argon having the lowest.

Chen *et al*. [23] reported the evaluation of *Cronobacter sakazakii* inactivation in non-fat dry milk powder by atmospheric cold plasma. In their study, 480 W of ACP treatment for 20-120 s led to 1.17-3.27 log CFU/g reduction of *C. sakazakii*. In addition, the authors observed that the log reduction value increased with increasing flow rate from 8 to 20 L/min. A similar tendency was also observed in the present study; as the nitrogen flow rate was increased, the log reduction of *E. coli* was increased. In the case of ACP with nitrogen gas runs no. 5 and 7, the mass and time were kept the same, while only the nitrogen gas flow rate increased from 0.04 L/min to 0.20 L/min, which caused the reduction to increase from 2.38 to 4.68 log CFU/g (Table 2). This high bactericidal effect according to an increased flow rate of gas has been reported in various studies [23, 24]. In addition, gram-negative bacteria are more sensitive to ACP treatment than gram-positive bacteria [25-27]. According to Kim *et al*. [28], nitrogen gas was the most effective for reducing *Aspergillus flavus* and *Bacillus cereus* spores in red pepper powder. Also, Sureshkumar *et al*. [29] reported that the addition of 2% O_2_ with nitrogen gas enhanced the bactericidal effect, with the presence of oxygen leading to the formation of nitric oxide and other reactive species.

### ANOVA Test and Second-Order Polynomial Model

The fitted second-order polynomial model was selected after conducting ANOVA based on the BBD. The BBD consists of three point levels for each factor, reducing total test runs without significantly sacrificing the accuracy [30]. The predictions from the response model and actual responses for the operating combinations from the BBD are shown in Table 2. The results of the ANOVA test of the models are shown in Table 3. As a result, the program derived the second-order polynomial equations (Eqs. 2-4) as follows through multiple regression analysis:

$$Argon=-3.4153-1.9040\times x_{1}+1.0753\times x_{2}+9.5744\times x_{3}-0.2015\times x_{1}\times x_{2}-7.2481\times x_{1}\times x_{3}+0.6803\times x_{2}\times x_{3}+1.8367\times x_{1}^{2}-0.0444\times x_{2}^{2}-25.6336\times x_{3}^{2}.$$ (2)

$$Air=2.6199-5.2265\times x_{1}+0.3478\times x_{2}-15.7279\times x_{3}-0.2393\times x_{1}\times x_{2}-2.0406\times x_{1}\times x_{3}+0.4645\times x_{2}\times x_{3}+3.0233\times x_{1}^{2}-0.0020\times x_{2}^{2}+62.3723\times x_{3}^{2}.$$ (3)

$$Nitrogen=1.3715-2.9802\times x_{1}+0.2388\times x_{2}+20.3173\times x_{3}-0.2501\times x_{1}\times x_{2}-12.3188\times x_{1}\times x_{3}+0.7360\times x_{2}\times x_{3}+2.3024\times x_{1}^{2}+0.0001\times x_{2}^{2}-31.7324\times x_{3}^{2}.$$ (4)

In the equations, *x*_1_, *x*_2_, and *x*_3_ refer to mass (g), time (min), and gas flow rate (L/min), respectively. The sum of squares, mean square, *F*-value, and *p*-value, as well as insignificant lack-of-fit values, accounted for the significance of the models (Table 3). Interestingly, the three factors of mass, time, and flow rate showed significantly different results in the ANOVA test according to the type of gases (Table 3). The *p*-values of mass factors of the three gases were less than 0.05, which means that mass had a significant effect on the models. In addition, the *p*-values for time were less than 0.05 for air and nitrogen gas; however, the *p*-value was not significant in the case of argon. The *p*-value of the argon gas model was 0.0526, which is slightly over 0.05. Only the nitrogen gas flow rate showed a significant effect as the *p*-value was less than 0.05, while the other gases, argon and air, did not. Furthermore, the “lack-of-fit” which describes the model compatibility was statistically significant for every independent variable. According to Kong *et al*. [31], the "*F*-value of Lack-of-Fit" implies the lack-of-fit is not significant relative to the experimental error. Also, the *p*-values for lack-of-fit were 46.98%, 29.55%, and 50.23%for argon, air, and nitrogen models, respectively. This means that there is a probability that such a large *F*-value occurs due to noise. Treatment time has been demonstrated in previous studies as being a significant parameter for bacterial reduction in *Staphylococcus aureus* and *E. coli* liquid media as well as foods including black pepper and sea bass fillet [32-35]. In addition, Niemira and Sites [24] reported that a gas flow rate of 40 L/min was more effective than 20 L/min in reducing bacterial viability. However, according to the results of the present study, only nitrogen flow rate showed a significant bactericidal effect during ACP treatment of wheat flour. This suggests that additional nitrogen gas will enhance bactericidal effect sufficiently.

### Optimization of ACP Treatment Using Three Gases

**Fig. 2:**
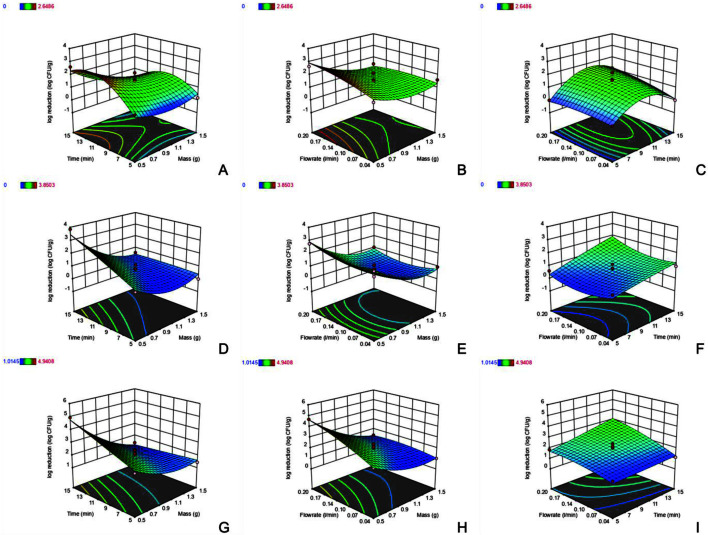
Response surface (3D) plots of the reduction values of *Escherichia coli* (log CFU/g) with three gases. Each row is arranged with three types of gas used. **A-C**, Argon; **D-F**, Air; **G-I**, Nitrogen. The figure order by row; the effect of time × mass, flow rate × mass, and flow rate × time.

The predicted and actual responses to the combined conditions of the BBD are shown in Table 2 for 3 types of gases and three variables. In addition, Fig. 2 shows a relationship between each factor and response value in the 3D plot.

**Argon**. The maximum reduction of *E. coli* after ACP treatment with argon gas was 2.65 log CFU/g for 0.5 g of wheat flour, 10 min of treatment time, and 0.20 L/min gas flow rate (Table 2). The *p*-value of mass × time was shown to be significant at 0.0451, meaning that ACP treatment using argon gas affects the microbial reduction as the flour mass decreases and the treatment time increases. For example, runs no. 3, 5, and 7, which had lower mass and longer treatment times, showed effective bactericidal results. However, the *p*-values of mass × flow rate and time × flow rate were over 0.05, which were not significant. These results mean that mass × flow rate and time × flow rate are not related to response value when performing ACP treatment with argon gas. Therefore, no bactericidal effect was observed in runs no. 4, 9, 10, and 11 (Table 2). We speculated that the mass, time, and flow rate, or their interactions, were not significant in these runs. In addition, Zhuang *et al*. [36] reported that mixtures of argon gas with other gases showed less bactericidal effects than other gas mixtures with oxygen, nitrogen, and carbon dioxide. In brief, these results about the argon gas model showed lower efficacy of bactericidal effect than other gases such as air and nitrogen.

**Air**. ACP treatment with air showed the maximum *E. coli* reduction of 3.85 log CFU/g with the conditions of 0.5 g, 15 min, and 0.12 L/min; whereas, no reduction was observed when the conditions of 1.5 g, 5 min, and 0.12 L/min (Table 2) were used. In the ANOVA, the significant coefficients were mass and time, and interaction coefficient of mass × time. The flow rate and its interaction coefficients were not significant. In the air gas model, the variation of mass and the time factor affected the reduction value. The minimum mass and maximum time had a greater influence on the reduction value, which was evidenced by runs no. 3, 5, and 7 (Table 3). The flow rate was not significant according to the *p*-value (0.355), but the interaction with mass and time was statistically significant. As in the current study, Mošovská *et al*. [33] found that ACP treatment with ambient air for 6 min reduced *E. coli* inoculated on 5 g of black pepper by more than 7.65 log CFU/g. Thus, ACP treatment with air has shown effectiveness for reducing *E. coli* in contaminated wheat flour; however, the flow rate of air used in the ACP treatment did not influence *E. coli* reduction.

**Nitrogen**. All of the coefficients in the nitrogen gas model were statistically significant and the most effective at reducing the number of viable *E. coli* among the three gases. The maximum and minimum log CFU/g reductions were 4.94 and 1.01 under conditions of 0.5 g, 15 min, and 0.12 L/min, and 1.5 g, 10 min, and 0.04 L/min, respectively. All of the coefficients such as mass, time, flow rate, and their interactions were shown to be statistically significant in the model unlike the other two types of gases. The flow rate of nitrogen gas had an effect on microbial reduction, which is shown in Table 2. In particular, runs no. 5 and 7 were programmed to the same 0.5 g of mass and 10 min of time, but the flow rate was programmed differently at 0.04 L/min and 0.20 L/min. As a result, when the flow rate was 0.04 L/min and 0.20 L/min the reduction value was 2.38 and 4.68 log CFU/g. Thus, the bactericidal effect of the nitrogen gas in ACP increased with flow rate, establishing that the flow rate of nitrogen gas significantly affects the reduction of *E. coli* in wheat flour.

Consistent with the current study, Jeon *et al*. [37] found that a gas mixture of 20% O_2_ : 80% N2 produced a higher bactericidal effect than using the ambient air. In addition, Patil *et al*. [38] found that a mixture of 10% O_2_ : 90% N2 in carrier gases showed a higher bactericidal effect on *Bacillus astrophaeus* spores in comparison to atmospheric air. Finally, Han *et al*. [39] reported that the nitrogen content in the treatment gas mixture generated reactive nitrogen species, which was the main cause of microbial inactivation.

### Validation of the Developed Models

The R^2^ value and the lack-of-fi*t* test were determined to check the significance of the selected models. Responses of variables were plotted in a 3D response surface to interpret the correlation among different combinations. Statistical values from developed models are shown in Table 4, which shows the fit statistics for the reduction value in the selected quadratic predictive model. The R^2^ values of models for argon, air, and nitrogen were 0.9060, 0.9217, and 0.9685, respectively, and corresponding to adjusted R^2^ values were 0.7852, 0.8210, and 0.9279. These results indicate a high level of correlation between the actual and predicted values in the model. Values of adequate precision for the three gases were higher than 4, confirming that the models were fitted. Zhuang *et al*. [36] reported that when most of the coefficients are statistically significant through RSM, the predicted results match with the actual results within the standard deviation bounds. Validation of a developed model for each gas was carried out with predicted solutions from the program, and two solutions were selected from each model. The results of the actual experiment through the derived solutions are shown in Table 5. As the result, all of the validation experiments were in statistical agreement with the predicted values. The results of an actual experiment were found to be within the standard deviations, the range with a significant level of 95% of the predicted value.

In this study we aimed to derive the response surface models for *E. coli* reduction in wheat flour using atmospheric cold plasma (ACP) with three types of gas. Simultaneously, the interactions between ACP treatment factors (mass, time, and flow rate) were evaluated. As a result, the most important factors of ACP treatment were shown to be the treatment factors mass and time. Interestingly, the interactions between factors differed by the type of gas used. The interactions of mass × time, and mass × flow rate using nitrogen gas were significantly effective on *E. coli* reduction but time × flow rate was not. Nitrogen gas was the most effective treatment gas on *E. coli* reduction in wheat flour using ACP treatment. In the validation test, ACP with nitrogen gas achieved the maximum *E. coli* reduction at the level of 5.55 log CFU/g. This study proposed ACP treatment models for reducing *E. coli* in wheat flour, and indicated that nitrogen was the most effective treatment gas after evaluation of three carrier gases. Lastly, the results of the present study have proposed the basic data for the industrial sterilization of wheat flour using ACP.

## Figures and Tables

**Table 1:** Experiment levels and ranges of independent variables designed to optimize the model according to the Box-Behnken design.

Variables	Symbols	Levels		

	Coded	-1	0	1
Mass (g)	*x*_1_	0.5	1	1.5
Time (min)	*x*_2_	5	10	15
Gas flow rate (L/min)	*x*_3_	0.04	0.12	0.20

**Table 2:** Box-Behnken design of three variables and response values (actual and predicted value means reduction value).

Coded symbols				Response (log CFU/g)					

				Actual			Predicted		

Run	*x*_1_	*x*_2_	*x*_3_	Argon	Air	Nitrogen	Argon	Air	Nitrogen
1	-1	-1	0	0.78	0.89	2.53	0.61	1.02	2.71
2	1	-1	0	0.19	0.00	1.43	0.50	0.40	1.60
3	-1	1	0	2.61	3.85	4.94	2.30	3.45	4.76
4	1	1	0	0.00	0.57	1.34	0.18	0.44	1.15
5	-1	0	-1	1.66	2.35	2.38	1.87	2.46	2.42
6	1	0	-1	1.61	0.96	1.01	1.34	0.80	1.05
7	-1	0	1	2.65	2.76	4.68	2.93	2.92	4.64
8	1	0	1	1.44	1.05	1.34	1.24	0.93	1.30
9	0	-1	-1	0.00	0.63	1.27	-0.03	0.39	1.06
10	0	1	-1	0.00	0.97	1.12	0.11	1.26	1.27
11	0	-1	1	0.00	0.60	1.85	-0.10	0.31	1.71
12	0	1	1	1.09	1.68	2.88	1.13	1.92	3.09
13	0	0	0	1.02	0.19	2.00	1.55	0.62	1.98
14	0	0	0	1.61	0.61	2.39	1.55	0.62	1.98
15	0	0	0	2.16	1.13	2.14	1.55	0.62	1.98
16	0	0	0	1.38	0.81	1.80	1.55	0.62	1.98
17	0	0	0	1.55	0.38	1.59	1.55	0.62	1.98

*x_1_*: wheat flour mass; *x_2_*: treatment time; *x_3_*: gas flow rate

**Table 3:** Results of ANOVA analysis for the quadratic models of argon, air, and nitrogen.

	d_f_	Sum of squares			Mean square			*F*-value			*p*-value		
			
		Ar	Air	N_2_	Ar	Air	N_2_	Ar	Air	N_2_	Ar	Air	N_2_
Model	9	11.54	14.65	19.84	1.28	1.63	2.2	7.5	9.16	23.9	0.0073**	0.004**	0.0002**
Mass		2.49	6.61	11.08	2.49	6.61	11.08	14.54	37.16	120.18	0.0066**	0.0005**	<0.0001**
Time		0.93	3.06	1.28	0.93	3.06	1.28	5.43	17.19	13.85	0.0526*	0.0043**	0.0074**
Flow rate		0.45	0.17	3.07	0.45	0.17	3.07	2.65	0.98	33.28	0.1475	0.355	0.0007**
Mass × Time		1.01	1.43	1.56	1.01	1.43	1.56	5.93	8.05	16.95	0.0451**	0.0251**	0.0045**
Mass × Flow rate		0.34	0.03	0.97	0.34	0.03	0.97	1.97	0.15	10.53	0.2037	0.7102	0.0142**
Time × Flow rate		0.3	0.14	0.35	0.3	0.14	0.35	1.73	0.78	3.76	0.2297	0.4074	0.0937*
Mass^2^		0.89	2.41	1.39	0.89	2.41	1.39	5.19	13.53	15.12	0.0568*	0.0079**	0.0060**
Time^2^		5.18	0.01	0	5.18	0.01	0	30.28	0.06	0	0.0009**	0.8121	0.9816
Flow rate^2^		0.11	0.67	0.17	0.11	0.67	0.17	0.66	3.77	1.88	0.4425	0.0932*	0.2124
Residual	7	1.2	1.24	0.65	0.17	0.18	0.09						
Lack of Fit	3	0.52	0.71	0.27	0.17	0.24	0.09	1.03	1.75	0.93	0.4698	0.2955	0.5023
Pure Error	4	0.68	0.54	0.38	0.17	0.13	0.09						
Cor Total	16	12.74	15.9	20.48									

**Significant with *p* < 0.05, *Significant with *p* < 0.1

**Table 4:** Statistical values obtained from the developed models for treatment gases.

Coefficient of determination	Argon	Air	Nitrogen
R^2^	0.9060	0.9217	0.9685
Adjusted R^2^	0.7852	0.8210	0.9279
Coefficient of Variation (%)	35.59	36.94	14.07
Standard Deviation	0.41	0.42	0.30
Adequate Precision	9.55	9.70	15.94

**Table 5:** Validation of the developed models for *E. coli* reduction in wheat flour using ACP treatment.

Gas	Derived solution			Actual condition			Response (log CFU/g)		Result
		
	x_1_	x_2_	x_3_	x_1_	x_2_	x_3_	Predicted	Actual	
Argon	-0.98	0.26	0.03	-1	0	0	2.66	2.91 ± 0.36	*Validated*
	1	0.05	-1	1	0	-1	1.64	1.50 ± 0.26	
Air	-1	1	1	-1	1	1	3.44	3.38 ± 0.27	*Validated*
	-1	0.99	-0.97	-1	1	-1	4.21	4.15 ± 0.68	
Nitrogen	-1	0.25	1	-1	0	1	4.94	4.96 ± 0.09	*Validated*
	-0.97	0.9	0.91	-1	1	1	5.63	5.55 ± 0.17	

*x_1_*: wheat flour mass; *x_2_*: treatment time; *x_3_*: gas flow rate
